# Traditional Cardiovascular Risk Factors Associated with Diagonal Earlobe Crease (Frank Sign) in Mexican Adults: Aging, Obesity, Arterial Hypertension, and Being Male Are the Most Important

**DOI:** 10.1155/2024/5598134

**Published:** 2024-06-21

**Authors:** Rogelio Molina-Gallardo, Nomely S. Aurelien-Cabezas, Daniel Tiburcio-Jimenez, Jorge E. Plata-Florenzano, Jose Guzman-Esquivel, Iram P. Rodriguez-Sanchez, Margarita L. Martinez-Fierro, Roque Molina-Osorio, Adrian A. De-la-Madrid-Cernas, Jorge Armando Barriguete-Melendez, Ivan Delgado-Enciso

**Affiliations:** ^1^Facultad de Medicina, Universidad de Colima, Colima, 28040, Mexico; ^2^Instituto Mexicano del Seguro Social, Delegación Colima, Villa de Álvarez, Colima, 28983, Mexico; ^3^Instituto Estatal de Cancerología, IMSS-Bienestar Colima, Colima, 28085, Mexico; ^4^Laboratorio de Fisiología Molecular y Estructural, Facultad de Ciencias Biológicas, Universidad Autónoma de Nuevo León, San Nicolás de los Garza, 66455, Nuevo León, Mexico; ^5^Unidad de Medicina Humana y Ciencias de La Salud, Universidad Autónoma de Zacatecas, Zacatecas 98160, Mexico; ^6^Universidad Anáhuac Online, Alcaldía Álvaro Obregón, C.P. 01840, Mexico

## Abstract

**Introduction:**

Cardiovascular risk factors such as obesity, type 2 diabetes, hypertension, smoking, and dyslipidemia enfold heart disease morbimortality. Diagonal earlobe crease has been proposed as a prognostic marker of extension and severity of illness in patients with acute coronary syndrome. But its usefulness remains unclear in patients with or without coronary disease.

**Methods:**

A case-control study was carried out on a total of 805 patients with and without cardiovascular risk factors or acute coronary syndrome. Univariate and multivariate binary logistic regression analyses were used to determine the probability of having diagonal earlobe crease with the presence of cardiovascular risk factors and acute coronary syndrome. Data were summarized as odds ratio with 95% confidence intervals and *P* values.

**Results:**

An unadjusted (univariate) analysis showed that being male, being older than 55 years, obesity, type 2 diabetes mellitus, arterial hypertension, smoking, and dyslipidemia, as well as having acute coronary syndrome, were associated with the presence of diagonal earlobe crease. The multivariate analysis showed that men (OR 1.6, 95% IC 1.1–2.4, *P*=0.007), being over 55 years old (OR 4.8, 95% IC 3.2–7.2, *P* < 0.001), being obese (OR 2.1, 95% IC 1.4–3.1, *P* < 0.001), having arterial hypertension (1.5, 95% IC 1.1–2.3, *P*=0.025), or suffering from acute coronary syndrome (OR 5.3, 95% IC 2.5–11.1, *P* < 0.001), were independent factors associated with diagonal earlobe crease. The rest of cardiovascular risk factors were not relevant in the multivariate model.

**Conclusions:**

In Mexican adults, having an acute coronary syndrome is not the only factor associated with diagonal earlobe crease but also being a man, older than 55 years, having high blood pressure and obesity. Diagonal earlobe crease may simply be caused by changes in the skin and connective tissues of the ears because of the aging process, obesity, and/or being male. These factors, by themselves, enfold cardiovascular risk due to well-known pathophysiological causes.

## 1. Introduction

Cardiovascular diseases (CVDs) are the leading cause of death worldwide [1–4]. CVDs include acute coronary syndrome, stroke, and peripheral arterial disease [1–4]. Meanwhile, cardiovascular risk factors (CVRF) are biological characteristics, lifestyle habits, or conditions that enfold the likelihood of morbimortality from cardiovascular disease (CVD) in the medium and long terms [5, 6].

Traditional cardiovascular risk factors include high blood pressure, type 2 diabetes mellitus, obesity, smoking, and dyslipidemia [5, 6]. Also, there are several biomarkers for the diagnosis and risk stratification of patients with CVD [7, 8], including clinical markers [9–11]. Diagonal earlobe crease (DELC) is a clinical sign first described by Frank [12]. DELC has been proposed as an early clinical marker of CVD, as well as a severity and prognosis factor of disease in acute coronary syndrome (ACS) [13–15]. In patients with CVD, DELC sign has been associated with CVRF, but the association of DELC with CVRF remains unclear.

It has been proposed that DELC could be used as a clinical marker to identify the subjects carrying cardiovascular risk factors with or without acute coronary syndrome (ACS).

## 2. Materials and Methods

### 2.1. Study Design and Ethical Aspects

A case-control (cross-sectional observational) study was carried out with 3 parallel groups. All procedures were performed according to the STROBE Statement [16] and the principles of the Helsinki Declaration. The study was approved by the local ethics and health research committee of IMSS, Colima, Mexico (Register: R-2016-601—24). During a routine medical consultation with the family doctor, the research project was explained to all participants, inviting them to participate voluntarily. An informed consent document was read to them, and after any questions were addressed, all participants signed the written informed consent in the presence of two witnesses.

### 2.2. Participants

From January 2016 to November 2017, patients from the outpatient consultation and hospitalization area of Hospital General de zona No. 1 IMSS were recruited. Patients were adults aged between 26 and 80 years old, with indistinct sex. Patients with partial or total congenital or acquired deformity of the external pinna were excluded. Participants with previous diagnosis of stroke, gestational diabetes, peripheral arterial disease, pulmonary disease, chronic kidney disease, infectious disease, and malignancy were excluded. Patients who decided to drop out of the study were eliminated.

Three groups were formed by systematic sampling of consecutive cases: control group (without CVRF, without CVD, and without history of ACS); cardiovascular risk factors' group (with one or more CVRF: type 2 diabetes mellitus, arterial hypertension, smoking, obesity, or dyslipidemia but without previous diagnosis or history of ACS at the beginning of the study); and acute coronary syndrome group: (with ACS at the beginning of the study but no previous ACS).

Acute coronary syndrome was classified as follows: non-ST elevation ACS (NSTE-ACS), which includes non-ST myocardial infarction (NSTEMI) and unstable angina. On the other hand, the ST-elevation ACS includes ST-elevation myocardial infarction (STEMI) [17]. A cardiovascular risk factor was defined as the presence of one or more of the following conditions: obesity, type 2 diabetes mellitus, arterial hypertension, smoking, or dyslipidemia. Type 2 diabetes mellitus was determined as the presence of a fasting blood glucose ≥126 mg/dl or two hours glycemia ≥200 mg/dl during an oral glucose tolerance test or a glycosylated hemoglobin (A1C) ≥6.5% or by the presence of classic symptoms of hyperglycemia or hyperglycemia crisis with a random glycemia ≥200 mg/dl [18] or by previous needs of hypoglycemic medication. Arterial hypertension was defined as the persistent elevation of blood pressure over ≥140/90 mmHg or the need for antihypertensive medication. Obesity was defined by a body mass index (BMI) equal or higher than 30 kg/m^2^ [19]. Dyslipidemia was defined by total cholesterol levels ≥200 mg/dl or triglycerides (TG) <150 mg/dl or by cholesterol bound to high-density lipoprotein (C-HDL) <40 mg/dl or by the need for lipid-lowering medication [20]. Smoking was defined as an active or former smoker with less than 15 years of smoking cessation (smoking index was not analyzed). DELC was defined as a diagonal lobe crease with more than 1 millimeter depth that extends obliquely from the tragus to the outer rim of the pinna lobe, covering at least two-thirds of the lobe length [14], without discontinuity. The unilateral or bilateral presence of DELC was considered positive for DELC.

### 2.3. Sample Size and Statistical Power

The sample size was calculated by a cross-sectional analysis to detect differences in diagonal earlobe crease (DELC) proportions between the two groups. The calculation was based on a study about diagonal earlobe crease and coronary artery disease in a Chinese population, which noted that the prevalence of DELC is higher in individuals with coronary artery disease determined by coronary artery angiography (75.2%) when compared to a population without coronary artery disease (46.2%) [14]. Forty-three patients from each group were required to reach the required power (0.8) when the statistical analysis was performed at the level of 2-tailed alpha (0.05). That calculation was made using a sample size calculator for two independent study groups with binomial primary endpoints.

### 2.4. Procedures

Patients were recruited from hospital as well as outpatient settings. ACS diagnosis and CVRF reports were made and registered in the clinical record by an emergency, cardiology, or internal medicine physician which were blinded to the study. Clinical information of each patient was obtained from the clinical record with prior informed consent. A directed interview in which participants reported previous medication, pathological, and smoking history was also carried out. Patients who met the selection criteria were divided as cases or controls in the study. Visual exploration of the earlobe was performed by a single trained physician with the patient in a sitting position with adequate lighting conditions and based on the previously described criteria. A total of 980 patients were recruited, 175 of which were excluded for not meeting the selection criteria, leaving a total of 805 patients (581 cases of CVRF, 68 cases of ACS, and 156 controls). The ratio was 10 : 37 : 4, controls for CVRF and ACS cases, respectively.

### 2.5. Statistical Analyses

In the descriptive analysis of the quantitative data, the mean was used as a measure of central tendency and standard deviation as a measure of dispersion. For qualitative data, frequencies and percentages were used. Normality plots of quantitative data were performed with the Kolmogorov–Smirnoff test. The comparison between the groups was carried out using the chi square test (*χ*^2^), the likelihood ratio chi-square test, and one-way analysis of variance (ANOVA). The association between the presence of CVRF or ACS with DELC was determined using odds ratios (OR) and 95% confidence intervals (95% CI) using univariate binary logistic regression analyses. Subsequently, significant predictors were added to the multivariate model, and with forward stepwise logistic regression, the most parsimonious model was identified. The probability used for the stepwise regression was set at 0.15 for variable input and 0.25 for removal. Spearman correlations were used for bivariate analyses (age vs. DELC, unilateral or bilateral). The area under the receiver operating characteristic (ROC) curve, confidence interval, cut-off point, sensitivity, and specificity of the age of the patients were calculated to predict the presence of DELC. Statistical tests were performed using SPSS version 21 software (IBM SPSS, Chicago, Illinois, USA). Statistical significance was considered a value of *P* < 0.05.

## 3. Results

A total of 805 patients (57% women and 43% men) were studied. The average age of the patients was 52.6 years. DELC was found in 36.77% of all patients. DELC was present in 77.94% of cases with ACS, in 38.38% of cases with CVRF without ACS, and in 12.82% of controls (Table 1). In all groups, the presence of bilateral DELC was more frequent than unilateral. Table 1 summarizes the main clinical characteristics of the participating subjects.

An association was found between the presence of DELC and the presence of at least one CVRF (OR = 2.0; 95% CI 1.6–2.6, *P* < 0.001) in subjects with no history of ACS, compared with subjects without any CVRF (control group). Otherwise, the presence of DELC was strongly associated with ACS, compared to the control group (OR = 24.0; 95% CI 11.4–50.4, *P* < 0.001) (Table 2). After the analysis was adjusted for age and sex, this association remained with an OR of 1.7 and 8.3 for FRCV and ACS, respectively (Table 2). Age was significantly correlated with the number of DELC in each patient (unilateral and bilateral). The older the patient, the more likely they are to have bilateral DELC (*r* = 0.123, *P*=0.034). The area under the ROC curve for age as a predictor of the presence of DELC was 0.76 (95% CI 0.72–0.79, *P* < 0.001, cut-off point 56 years). Age of >56 showed a sensitivity of *a* = 0.69 and a specificity of *a* = 0.72.

The analysis for each cardiovascular risk factor in the univariate model showed that all were associated with the presence of DELC (Table 3). However, the multivariate analysis of the present study showed that men (OR 1.6, 95% IC 1.1–2.4, *P*=0.007), over 55 years old (OR 4.8, 95% IC 3.2–7.2, *P* < 0.001), obese (2.1, 95% IC 1.4–3.1, *P* < 0.001), with arterial hypertension (1.5, 95% IC 1.1–2.3, *P*=0.025), or with previous ACS (OR 5.3, 95% IC 2.5–11.1, *P* < 0.001), were independent factors associated with DELC. The rest of CVRFs were not significative in the multivariate model (Table 3). Among patients who had DELC (unilateral or bilateral), obesity, arterial hypertension, ACS, or male sex were not associated with having bilateral DELC more than unilateral (data not shown). However, individuals 56 years of age or older were 1.6 times more likely to have bilateral than unilateral DELC, compared with subjects of 55 years or younger (crude OR 1.65, IC 95% 1.01.2.76, *P*=0.038).

## 4. Discussion

Hypertension and obesity are the main CVRF associated with DELC, although its association is stronger with ACS. Male sex and age over 56 years were also factors strongly associated with DELC. Previous studies have shown the relationship between ACS and DELC [21, 22]. However, studies on the association of DELC with CVRF as independent factors to ACS are still scarce.

In contrast to the present investigation, most investigations have studied CVRF and their association with DELC within populations with ischemic cardiovascular disease [23–25]. A previous study conducted in patients with myocardial infarction found significant associations of DELC with arterial hypertension (*P* < 0.001) and diabetic retinopathy (*P* < 0.05) but not with smoking and hypercholesterolemia [23]. In another study that included patients with coronary artery disease diagnosed by computed tomography coronary angiography, it was observed that subjects with DELC had a higher prevalence of arterial hypertension and hyperlipidemia than subjects without DELC [24]. In the presence of other noncoronary vascular diseases, a high frequency of DELC has been observed in patients with acute ischemic stroke with arterial hypertension and diabetes mellitus, compared to the group that does not present the equal CVRF (*P* < 0.001) [25].

Some research has shown the relationship between DELC and CVRF. In the Pakistani and South Asian population, the association of DELC with coronary artery disease was reported [26], also showing that patients with arterial hypertension and diabetes mellitus, but not smokers, had DELC more frequently [26]. Another study showed that DELC was associated with coronary artery disease, hypertension, or smoking but not diabetes mellitus, dyslipidemia, and obesity [27]. It has been proposed that the simultaneous presence of more than 3 CVRF increases significantly more the frequency of DELC among subjects with coronary artery disease than in those without coronary artery disease [28].

Meanwhile, in populations free of cardiovascular disease, some studies have explored the relationship between DELC and CVRF [29–31]. For example, in adult subjects with DELC free of atherosclerotic disease, the presence of DELC was significantly correlated with the body mass index (*r* = 0.178, *P*=0.044) and arterial hypertension (*r* = 0.233, *P*=0.008) [29]. Besides, in Korean patients, it has been found that regardless of age, DELC presents a significant association with metabolic syndrome in the absence of coronary artery disease (OR = 1.88; CI = 1.62–2.17 and OR = 2.05; CI = 1.67–2.52, men and women, respectively) [32].

Despite these results, some populations exhibit contradictory results: a study carried out in Turkey indicates that in the presence of DELC, patients with asymptomatic hypertension but no coronary disease present a higher frequency of diabetes mellitus (*P*=0.02), smoking (*P*=0.002), and a higher BMI (*P*=0.004) as well as a significant difference in the values of the cardio-ankle vascular index [30]. Nevertheless, another investigation in the same population shows that patients without coronary artery disease have no significant differences between subjects with and without DELC with respect to arterial hypertension, diabetes mellitus, smoking, BMI, low-density lipoprotein cholesterol (LDL-C), cholesterol bound to high-density lipoproteins (HDL-C), and TG [33]. In this sense, even though subjects with DELC and without cardiovascular disease have higher epicardial adipose tissue (EAT) and carotid intimal media thickness (CIMT) than subjects without DELC [29, 33] and both EAT and CIMT are associated with coronary disease [34, 35], it was not found that EAT and CIMT present a significant correlation with BMI, HDL-C, LDL-C, and TG [33]. Finally, an investigation carried out in the USA found no relationship between DELC and CVRF: arterial hypertension, diabetes mellitus, smoking, weight, and hypercholesterolemia [36].

As can be seen, the associations found between CVRF and DELC vary between different studies, which can be attributed to the differences between the several methodologies used and the populations analyzed. In most studies, including the present one, a strong association between DELC and coronary artery disease is maintained, with arterial hypertension being the CVRF most frequently associated with DELC in the different populations analyzed [26, 27]. Some authors have documented that the presence of bilateral DELC has an odds' ratio like that found for traditional cardiovascular risk factors such as arterial hypertension, diabetes mellitus, and hypercholesterolemia in cardiovascular diseases [37]. In fact, objective and quantitative systems have been proposed for the evaluation of the characteristics of DELC and achieve its effective identification in cardiovascular diseases [38], and hence, this topic continues to be important.

Furthermore, it is important to mention that in the present study, even when the participants from the control group were significantly younger than participants of ACS and CVRF groups, this difference between the groups were statistically adjusted by multiple regression analysis. This adjustment allowed to evaluate the effect of each variable on the result independently; therefore, this variation did not affect the finding of association [39]. Also, a strength of the present study is that it shows that the age of ≥56 years is as associated with DELC at a similar level as its association with ACS. The association of age ≥56 years remained even after adjusting for the presence of ACS or CVRF, confirming that age was independently associated with these conditions. Although it has been previously reported that age does not seem to influence in the presence of these conditions [34], the high frequencies of DELC in people older than 60 years (48%) have been previously reported, and this finding is compatible with the hypotheses that have indicated that their presence could be related to the effects of aging [40].

Similarly, it is important to mention that men were more likely to have a DELC compared to women, a finding that has only been reported recently: Wang et al. report that in the group with DELC had the highest male/female ratio (M/F: 32/13) compared to controls without DELC (M/F: 24/21) [41]. A previous report mentioned that looking old for your age is a marker of poor cardiovascular health in both men and women [9]. Besides, it has been previously described that men usually have more signs of aging, and this is likely to be partly explained by the physiological higher aging effect over the male skin due to decline in collagen synthesis secondary to the decrease in androgen levels [42, 43]. This is relevant information since during the physical examination of a patient, although finding a DELC may be indicative of cardiovascular risk, it may also simply be due to aging, especially in men.

The present study has some limitations because we did not analyze the pathophysiological mechanisms by which DELC is associated with each cardiovascular risk factor. We could hypothesize that DELC is related to changes in the skin and connective tissue in the ears, produced simply by aging, obesity, and/or being male. These factors, by themselves, enfold cardiovascular risk due to well-known pathophysiological causes.

## 5. Conclusions

In Mexican adults, having an ACS is not the only factor associated with DELC but also being a man, older than 55 years, having arterial hypertension and obesity.

## Figures and Tables

**Table 1:** Main clinical characteristics of the participating subjects.

	All	Control	ACS	CVRF	*P*
	*n* 805 (%)	*n* 156 (%)	*N* 68 (%)	*N* 581 (%)	
Age	52.60 ± 16.5	42.2 ± 16.1	64.2 ± 15.3	54.0 ± 15.3	<0.001^*∗*^
Sex					0.001^*∗∗*^
Female	459 (57.0%)	103 (66.0%)	27 (39.7%)	329 (56.6%)	
Male	346 (42.9%)	53 (33.9%)	41 (60.3%)	252 (43.3%)	
Obesity	347 (43.1%)	—	25 (36.7%)	322 (55.4%)	0.004^*∗∗∗*^
Diabetes mellitus	224 (27.8%)	—	33 (48.5%)	191 (32.8%)	0.015^*∗∗∗*^
Hypertension	275 (34.1%)	—	48 (70.5%)	227 (39.0%)	<0.001^*∗∗∗*^
Smoking	195 (24.2%)	—	28 (41.1%)	167 (28.7%)	0.049^*∗∗∗*^
Dyslipidemia	329 (40.8%)	—	36 (52.9%)	293 (50.4%)	0.025^*∗∗∗*^
DELC^†^	296 (36.7%)	20 (12.8%)	53 (77.9%)	223 (38.3%)	<0.001^*∗∗*^
Unilateral DELC	113 (14.0%)	11 (7.1%)	17 (25.0%)	85 (14.6%)	
Bilateral DELC	182 (22.6%)	9 (5.8%)	36 (52.9)	137 (23.6%)	

^†^DELC: presence of diagonal earlobe crease, unilateral, and bilateral. ^*∗*^ANOVA test between cardiovascular risk factors (CVRF), acute coronary syndrome (ACS), and control groups. ^*∗∗*^Likelihood ratio chi-square test that included CVRF, ACS, and control groups. ^*∗∗∗*^Chi-square test, including ACS and CVRF groups. Age in years ± standard deviation.

**Table 2:** Diagonal earlobe crease (DELC) association adjusted for age and sex strata.

Groups	DELC		cOR	95% C.I.	*P* value	aOR	95% C.I.	*P* value
	No (%)	Yes (%)						
Control	87.2	12.8	Reference					
CVRF	61.6	38.4	2.05	1.60–2.64	**<0.001**	1.73	1.33–2.25	**<0.001**
ACS	22.1	77.9	24.02	11.45-50.40	**<0.001**	8.31	3.76-18.37	**<0.001**

DELC: diagonal earlobe crease, unilateral, and bilateral. cOR: crude odds ratio, aOR: odds ratio adjusted for confounding variables (sex: male/female and age strata ≤55 years and ≥56 years). *P* < 0.05 means statistically significant and is marked in bold. CI: confidence interval. CVRF: cardiovascular risk factors. ACS: acute coronary syndrome. Individuals without CVRF and ACS were set as the reference (control) group for association analysis.

**Table 3:** Univariate and multivariate logistic regression analyses of factors associated with the diagonal earlobe crease (DELC).

Variable	DELC %		Univariate model			Multivariate model		
	No	Yes	cOR	95% C.I.	*P*	aOR^*∗*^	95% C.I.	*P*
Age								
≤55 years	81.0	19.0	Reference					
≥56 years	41.4	58.6	6.04	4.40–8.28	<0.001	4.87	3.29–7.23	<0.001
Sex								
Women	68.6	31.4	Reference					
Men	56.1	43.9	1.71	1.28–2.29	<0.001	1.66	1.15–2.41	0.007
Obesity								
No	68.1	31.9	Reference					
Yes	56.8	43.2	1.62	1.21–2.17	0.001	2.14	1.46–3.13	<0.001
Diabetes mellitus								
No	68.3	31.7	Reference					
Yes	50.0	50.0	2.15	1.57–2.95	<0.001			
Hypertension								
No	73.2	26.8	Reference					
Yes	44.0	56.0	3.47	2.56–4.72	<0.001	1.57	1.06–2.33	0.025
Smoking								
No	66.2	33.8	Reference					
Yes	53.8	46.2	1.68	1.21–2.33	0.002			
Dyslipidemia								
No	69.0	31.0	Reference					
Yes	54.1	45.9	1.89	1.37–2.60	<0.001			
ACS								
No	67.0	33.0	Reference					
Yes	22.1	77.9	7.18	3.96-13.00	<0.001	5.35	2.57–11.11	<0.001

DELC: diagonal earlobe crease, unilateral, and bilateral. cOR: crude odds ratio, aOR: adjusted odds ratio. ^*∗*^ Adjusted for the covariates of age (≤55 and ≥56 years), sex, obesity, hypertension, and acute coronary syndrome (ACS). *P* < 0.05 means statistically significant. CI: confidence interval. Important variables in the univariate model were subsequently added to the multivariate model and a forward stepwise logistic regression identified the most parsimonious model. The probability used for the stepwise regression was set at 0.15 for entry of variables and 0.25 for removal.

## Data Availability

All relevant data appear in this study. The datasets used and/or analyzed in the current study are available from the corresponding author upon reasonable request.
